# Surgical Management of Life-Threatening Thyroid Haematoma following Occult Blunt Neck Trauma

**DOI:** 10.1155/2016/4307695

**Published:** 2016-10-25

**Authors:** Ronak Ved, Neil Patel, Michael Stechman

**Affiliations:** University Hospital of Wales, Cardiff CF14 4XW, UK

## Abstract

A 42-year-old man arrived at the emergency department in severe respiratory distress, requiring immediate intubation and ventilation. An emergency computed tomography (CT) neck scan identified a substantial haematoma within a multinodular goitre, necessitating an emergency total thyroidectomy. It was later discovered that the patient had been the victim of an assault involving blunt trauma to the anterior neck. Five days postoperatively the patient was extubated and was well enough to self-discharge the following day. Pathology revealed the lesion to be a ruptured follicular adenoma within his multinodular goitre. Signs of this rare but life-threatening condition may be subtle on initial presentation, particularly if the patient is obtunded. Patients with suspected blunt neck trauma should be observed for signs of respiratory distress. If this develops, the patient should be intubated to facilitate CT scan, and if thyroid haematoma is confirmed, emergency thyroidectomy is the definitive treatment.

## 1. Introduction 

In cases of blunt trauma involving the neck, a high index of suspicion is warranted for thyroid haematoma, as signs may not be present on initial examination. We report a case of occult blunt injury to the neck of a 42-year-old homeless male patient with a preexisting multinodular goitre (MNG). Invasive airway management, cross-sectional imaging, and emergency thyroidectomy were necessary.

## 2. Case Presentation

The patient was admitted by ambulance after workers in a hostel observed him develop gradual onset difficulty of breathing and swelling of the throat. There was no other history available at this time. He arrived at the emergency department in severe respiratory distress and with fluctuating consciousness. His vital signs at presentation were respiratory rate of 28 breaths per minute, heart rate of 110 beats per minute, 100% saturation on 100% O_2_, BP 170/109, and temperature 37.4°. His neck was markedly swollen and tender, necessitating immediate nasotracheal intubation and ventilation. He was then taken for emergency computed tomography (CT) of the neck and thorax to determine the cause. The patient had an established multinodular goitre, took 200 mcg levothyroxine daily, had no known allergies, and was an alcoholic. Admission biochemistry demonstrated no evidence of hyperthyroidism with TSH 5.52 mU/L (0.30–4.40) and fT4 12.3 pmol/L (9.0–19.1). Neither anti-TPO nor TSH-R antibody titres were elevated.

**Figure 1:**
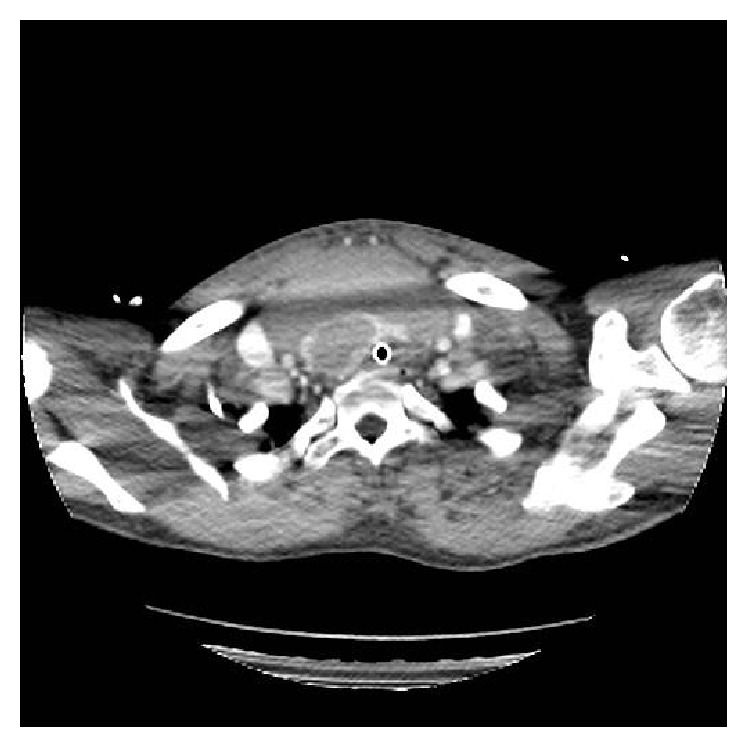
Arterial phase CT neck: axial view of neck haematoma demonstrating compression of the intubated trachea.

**Figure 2 fig2:**
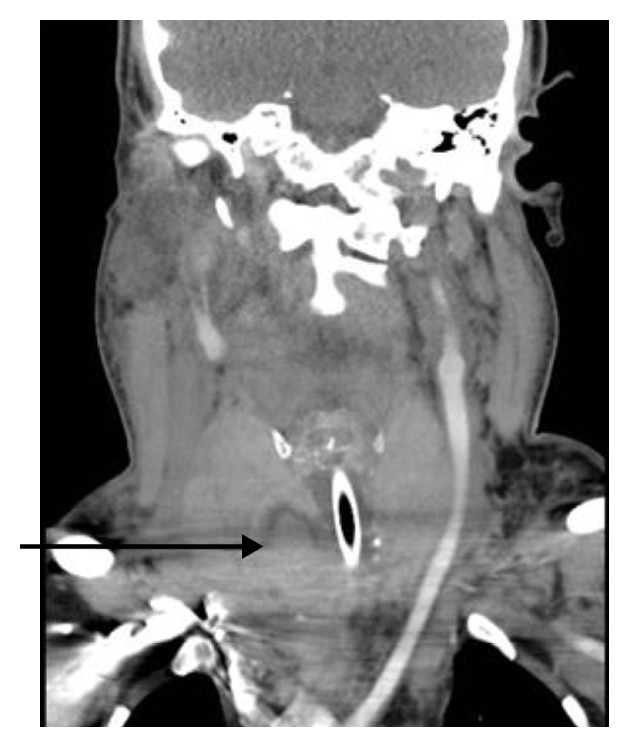
Arterial phase CT neck: coronal view of neck haematoma demonstrating lateral displacement of the great vessels and trachea and hyperdense haemorrhage within right-sided thyroid nodule (arrow).

**Figure 3:**
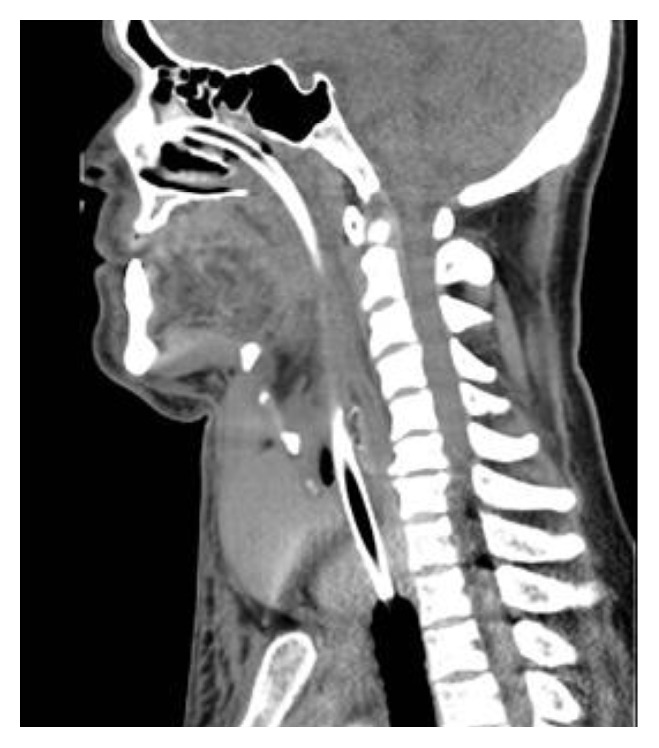
Arterial phase CT neck: parasagittal view of neck haematoma.

Whilst being transferred to the CT scanner he was commenced on empirical intravenous antibiotics and steroids. A substantial neck collection and multinodular goitre were identified on the arterial phase neck CT. The 3.5 × 10 × 10 cm (anterior-posterior × lateral × superior-inferior) mass was anterior to the thyroid and displaced the trachea to the left. The great vessels were displaced laterally by the collection but remained undamaged (Figures 1 [Fig fig2]–3).

The thyroid gland was noted to contain multiple cystic structures, and one of these in the right lobe was of increased density (Figure 3) suggesting haemorrhage within that nodule. A direct connection was strongly suspected between this haemorrhagic lesion and the anterior neck collection.

**Figure 4:**
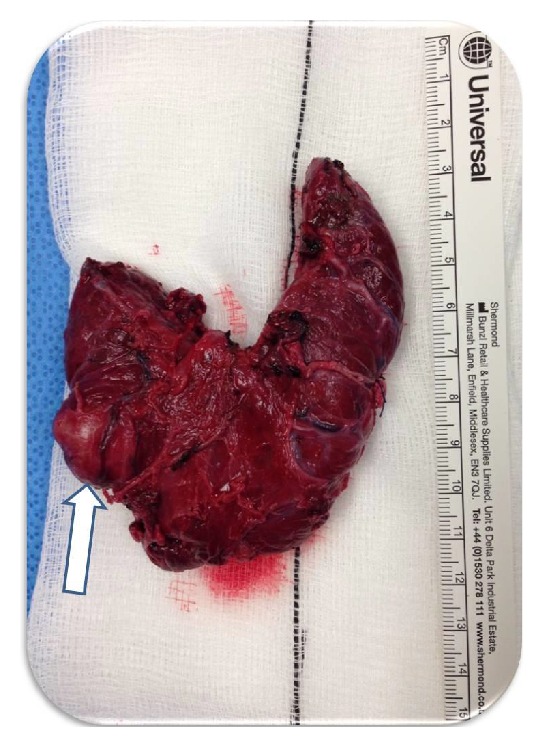
Postoperative specimen of the goitrous right lobe of the thyroid gland, with the right-sided nodule clearly visible adjacent to the isthmus (white arrow), from which the pulsatile arterial haemorrhage was identified intraoperatively.

This necessitated an emergency total thyroidectomy. The cause was identified as an arterial vessel within the wall of a ruptured right-sided thyroid nodule (Video 1 in Supplementary Material available online at http://dx.doi.org/10.1155/2016/4307695). A right thyroid lobectomy was performed after evacuation of the haematoma. A left lobectomy was also carried out as persistent bleeding originating from the enlarged left lobe, which may have been related to venous bleeding within the lobe caused by the blunt neck injury. The nodule is visible in Figure 4.

During the patient's convalescence on the intensive care unit postoperatively, it was discovered that he had been the victim of an assault, involving blunt trauma to the anterior neck a few hours before his arrival in the emergency department. Pathology results revealed the lesion to be a ruptured 35 mm follicular adenoma within a multinodular goitre, without any features of papillary nuclear features nor any evidence of capsular or vascular invasion. The nodule was expanding from the lower right pole into the isthmus (Figure 4). A number of small follicular adenomas were identified in the left lobe of the thyroid gland. However, none of these were as enlarged as the right-sided nodule responsible for the arterial bleeding.

Flexible nasendoscopy on the 3rd postoperative day revealed moderate supraglottic oedema. Intravenous dexamethasone and antibiotics were therefore continued until 5 days postoperatively, when repeat visualisation of the laryngopharynx revealed resolution of the oedema. The patient was successfully extubated and converted to oral antibiotics and steroids. His recovery thereafter was rapid, and on the 6th postoperative day he was well enough to self-discharge from hospital. He did not attend arranged follow-up with endocrine surgery but has since been reviewed by local internal medicine and substance misuse teams, and there have been no further concerns regarding his neck.

## 3. Discussion 

Neck trauma is common. However secondary haemorrhage of the thyroid gland as a result of trauma is rare [2]. Goitrous glands, with their increased size and vascularity, carry an increased risk for posttraumatic haemorrhage [3]. Haemorrhage into a normal gland has also been reported [4].

Symptoms of traumatic thyroid haematoma may not be apparent immediately after a neck injury. The trauma may be direct impact, but cervical hyperflexion/extension, deceleration injuries, and even Valsalva manoeuvres can all cause thyroid haemorrhage [3]. The onset of airway compromise is also variable: it may develop within minutes [4], days [5], or not at all [6]. Other than frank airway compromise and respiratory distress, symptoms of thyroid haematoma include a painful neck mass, dysphagia, and hoarseness [5]. Clinical assessment can be difficult, particularly if there is incomplete history or low GCS, as in this case. Therefore, a high index of suspicion is required if there is any indication of neck trauma, with or without a palpable neck mass.

Radiological investigations can establish the diagnosis of thyroid gland injury. Emergency arterial phase CT scanning was the investigation of choice in our case as the patient presented with rapid onset respiratory distress out of hours; a CT scan permitted assessment for both thyroid haemorrhage and great vessel injury within the neck. Ultrasound has been utilised as a diagnostic tool in cases without rapid onset of airway compromise [5]. Fiberoptic laryngoscopy can also prove helpful in these stable cases to rule out laryngeal injury, as this may not be demonstrated on imaging [4].

Thyrotoxicosis has been reported after thyroid trauma [3], although it is not universal, as illustrated by the absence of hyperthyroidism in our case. Thyroid function should therefore be checked if there is suspicion of thyroid injury, even in the absence of a palpable neck mass. Given the increased risk of thyroid haemorrhage for patients with goitres, which may potentially be undiagnosed until the event, appropriate investigations to determine causes of hyper/hypothyroidism should be undertaken if a traumatic thyroid haematoma is identified.

**Figure 5:**
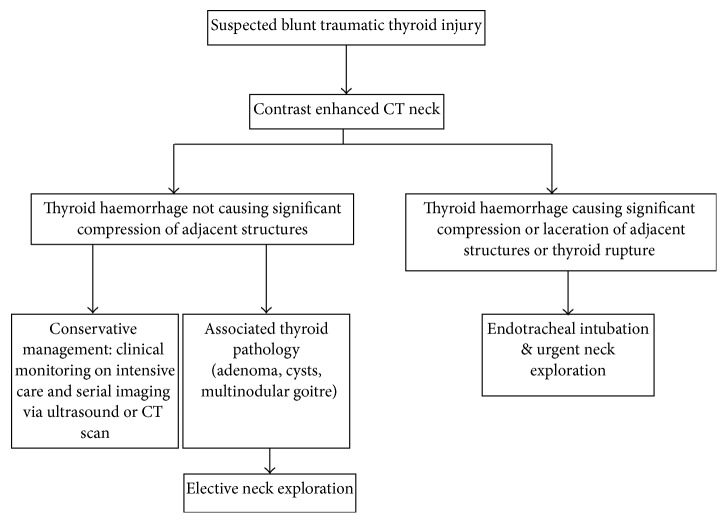
Adapted algorithm for the assessment and management of suspected thyroid haematoma from Heizmann et al. [1].

Traumatic thyroid haematomas may be successfully managed both surgically and conservatively [3]. Increasing size and/or suspicion of airway compromise are indications for immediate intubation, imaging, and neck exploration. Signs of respiratory distress were readily apparent in our case, which necessitated emergency surgical intervention. Heizmann et al. proposed a simple algorithm to aid in the investigation and treatment after blunt neck trauma [1]. This algorithm advocates CT neck for all patients with suspected blunt neck injury, after which the severity of any thyroid injury then dictates the requirement for urgent neck exploration (Figure 5). The pathway also suggests elective thyroidectomy for patients with minor haemorrhage in the presence of structural thyroid abnormalities, owing to the established association between significant bleeding and thyroid disease. This is a useful paradigm for management of suspected thyroid trauma; however it is dependent upon an index of suspicion for thyroid injury in the first instance, which may not be obvious in the absence of a clear history of trauma, as in our case. It is also imperative that airway stability is obtained prior to CT neck scanning if necessary and that there should be a low threshold for patients following the conservative or elective surgical management pathway to be reviewed for urgent surgical intervention, as life-threatening features of an expanding neck haematoma can evolve rapidly.

This algorithm does not specify which procedures should be performed if thyroid haemorrhage is identified at neck exploration. In the presented case a pulsatile arterial haemorrhage was identified from an individual right-sided thyroid nodule, and therefore a hemithyroidectomy could have been performed in the first instance. However, we elected to perform a total thyroidectomy to (a) obviate the risk of continued, delayed bleeding from the enlarged left lobe, which was noted intraoperatively, and (b) avoid the potential need for reoperative left hemithyroidectomy in the future in an already goitrous patient, who was known to regularly fail to attend medical follow-up appointments. The decision between hemi- and total thyroidectomy in these cases may often need to be made intraoperatively, after evacuation of the haematoma when macroscopic assessment of likely bleeding sources from the thyroid gland can be carried out.

The presentation of thyroid haematoma may be subtle or delayed, and in these cases substantial thyroid haematomas may be missed [3–5]. Such cases could rapidly evolve into life-threatening scenarios. Regular and comprehensive monitoring of airway and breathing is paramount if conservative management is contemplated.

## 4. Clinical Significance

Cases of blunt neck trauma should be observed meticulously for signs of respiratory distress in the first instance. If this develops, the patient should be intubated to facilitate CT scan. If thyroid haematoma is confirmed, emergency thyroidectomy is the definitive treatment. There must be a low threshold for these interventions if a conservative approach is adopted.

## Supplementary Material

Video 1: Intraoperative video demonstrating the right-sided thyroid nodule responsible for the arterial bleeding.
